# Magnesium depletion score and stroke in US adults: Analysis of NHANES 1999 to 2018

**DOI:** 10.1097/MD.0000000000046376

**Published:** 2025-12-19

**Authors:** Li-Chao Zhang, Zhi-Qiang Ye, Wen-Liang Shuai, Chen-Qi He, Xu-Ying Yu, Li-Min Zhuang, Li-Min Gong

**Affiliations:** a Department of General Practice, The Second Affiliated Hospital, Jiangxi Medical College, Nanchang University, Jiangxi, China; b Department of Cardiovascular Medicine, The Second Affiliated Hospital, Jiangxi Medical College, Nanchang University, Jiangxi, China.

**Keywords:** cardiovascular risk, magnesium deficiency, magnesium depletion score, NHANES, public health intervention, stroke

## Abstract

Stroke remains a leading cause of death and disability globally, with substantial socioeconomic burdens. Magnesium is essential for cardiovascular and neurological homeostasis, but serum magnesium levels alone inadequately reflect systemic magnesium status. The Magnesium Depletion Score (MDS), a composite metric integrating diuretic use, proton pump inhibitor PPI use, renal function (estimated glomerular filtration rate [eGFR]), and alcohol consumption, provides a more comprehensive assessment of magnesium deficiency. This cross-sectional study analyzed 10,136 US adults from the National Health and Nutrition Examination Survey (1999–2018). MDS was calculated based on 4 components, and weighted multivariable logistic regression models were used to evaluate the association between MDS and stroke risk. Restricted cubic spline analysis was applied to assess dose-response relationships. Higher MDS scores were independently associated with increased stroke risk. After full adjustment for confounders (demographics, lifestyle factors, and cardiovascular comorbidities), each 1-unit increase in MDS was linked to a 27% higher odds of stroke (OR = 1.27, 95%CI: 1.12–1.44). Restricted cubic spline analysis demonstrated a linear dose-response relationship without evidence of a threshold effect. Systemic magnesium depletion, as measured by MDS, is significantly associated with stroke risk. MDS may serve as a practical tool for identifying individuals at elevated risk, underscoring the importance of targeted nutritional interventions and lifestyle modifications in stroke prevention.

## 1. Introduction

Stroke, caused by obstruction or rupture of cerebral blood vessels, leads to damage or necrosis in specific brain regions, resulting in long-term neurological dysfunction, disability, and even death. This imposes a substantial socioeconomic burden and strains healthcare systems.^[1]^ As a major public health concern in the United States, stroke accounts for over 795,000 new cases annually, including approximately 610,000 first-time strokes and 185,000 recurrent strokes.^[2]^ Reducing stroke incidence and advancing prevention strategies are therefore of paramount importance. In recent years, trace elements – particularly magnesium, zinc, and selenium – have garnered attention for their potential roles in stroke pathogenesis through various biological mechanisms.^[3–5]^

Magnesium, the second most abundant divalent cation in the human body, is involved in over 300 enzymatic reactions.^[6]^ Despite a recommended daily intake of 310 to 420 mg for adults, approximately 60% of American adults fail to meet this requirement.^[7]^ Magnesium deficiency impacts multiple systems: in the nervous system, it regulates central excitability, with deficiency linked to neuronal hyperactivity, anxiety, and stress.^[8]^ In the cardiovascular system, adequate magnesium promotes vascular smooth muscle relaxation. Conversely, magnesium insufficiency may lead to vasoconstriction, hypertension, and increased risks of atherosclerosis and arrhythmias^[9,10]^; metabolically, it maintains bone health via calcium/vitamin D regulation and insulin sensitivity, with deficiency impairing bone formation and increasing diabetes risk.^[11–13]^ Epidemiological studies show an inverse association between dietary magnesium intake and stroke risk, with the highest intake group demonstrating a 44% lower risk compared to the lowest.^[14]^

Previous research has focused on serum magnesium levels, but these do not fully reflect systemic magnesium status.^[15]^ The Magnesium Depletion Score (MDS), a novel tool integrating diuretic use, proton pump inhibitor (PPI) use, estimated glomerular filtration rate (eGFR), and alcohol consumption, provides a comprehensive assessment of magnesium deficiency – higher scores indicate more severe depletion.^[16]^ No study has yet evaluated MDS in relation to stroke risk. Using 1999–2018 NHANES data on 10,136 participants, this study investigates the association between MDS and stroke risk to inform public health interventions.

## 2. Materials and methods

### 2.1. Study population and design

**Figure 1.**
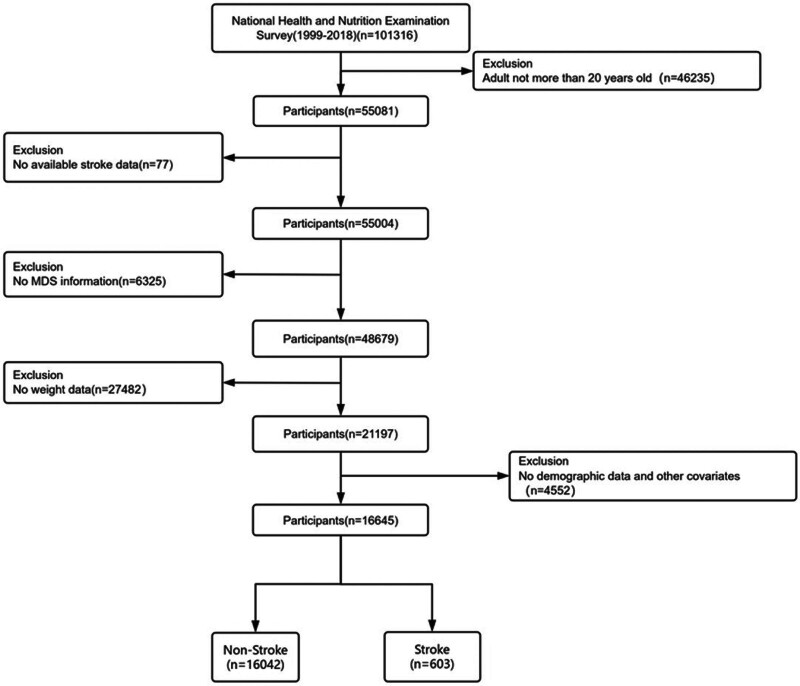
Flowchart of participant selection from National Health and Nutrition Examination Survey 1999 to 2018.

Data were obtained from the NHANES website (https://www.cdc.gov/nchs/nhanes/index.html). Conducted by the CDC, NHANES is a nationally representative, stratified, multistage probability survey. Ten cycles (1999–2018) were included, covering demographic, interview, and examination data. All participants provided informed consent, and the study was approved by the National Center for Health Statistics Ethics Review Board.^[17,18]^ Authors cannot access to information that could identify individual participants during or after data collection. From an initial sample of 101,316 adults, exclusions included: age ≤ 20 years (n = 46,235), missing stroke data (n = 77), missing MDS scores (n = 6325), missing weight data (n = 27,482), and missing covariates (n = 4552). The sample selection process is illustrated in Figure 1.

## 2.2. Magnesium depletion score calculation

MDS assesses systemic magnesium deficiency via 4 indicators: 1) Diuretic use: 1 point for current use, 0 otherwise. 2) PPI use: 1 point for current use, 0 otherwise. 3) eGFR: 1 point for 60 to 90 mL/min/1.73 m², 2 points for <60 mL/min/1.73 m². 4) Alcohol consumption: 1 point for >1 drink/day (women) or >2 drinks/day (men) (1 drink = 14 g ethanol). Participants were categorized into 6 groups: MDS = 0, 1, 2, 3, 4, or 5.

## 2.3. Stroke diagnosis

Stroke history was self-reported via the Medical Conditions Questionnaire: “Has a doctor ever told you that you had a stroke?” Participants answering “yes” were classified as stroke cases.

## 2.4. Covariate assessment

Demographics (age, sex, race/ethnicity, education, marital status, income, poverty index) were self-reported. Weight and height were measured by trained technicians, with body mass index (BMI) calculated as kg/m². Diabetes was defined per International Diabetes Federation criteria^[19]^: fasting glucose ≥ 7.0 mmol/L, post-glucose load ≥ 11.1 mmol/L, antidiabetic medication use, or self-reported diagnosis. Hypertension was defined as systolic blood pressure ≥ 140 mm Hg, diastolic blood pressure ≥ 90 mm Hg, or antihypertensive medication use. Coronary heart disease (CHD) was self-reported physician-diagnosed.

## 2.5. Statistical analyses

Baseline characteristics were described using means ± SD for continuous variables and percentages for categorical variables, analyzed via linear regression and chi-square tests, respectively. Weighted univariable and multivariable logistic regression models evaluated associations between MDS and stroke, with 4 models constructed: crude model: Unadjusted. Model 1: Adjusted for age, sex, race/ethnicity, marital status, income, and education. Model 2: further adjusted for BMI, alanine aminotransferase (ALT), aspartate aminotransferase (AST), triglycerides (TG), HDL cholesterol (HDL-C), total cholesterol (TC), LDL cholesterol (LDL-C), smoking, and alcohol consumption. Model 3: Additionally adjusted for diabetes, hypertension, and CHD. Restricted cubic spline (RCS) analysis explored dose-response relationships, and stratified analyses by sociodemographic and lifestyle factors were performed. All analyses accounted for survey weights, with *P* <.05 considered significant. Analyses were conducted using R 4.4.2.

## 3. Results

### 3.1. Baseline characteristics by stroke history

Table 1 compares the baseline characteristics of the study population stratified by stroke history. Individuals with a history of stroke exhibited distinct demographic and clinical profiles compared to those without stroke. Specifically, stroke cases were more likely to be older, identify as non-Hispanic White, be smokers, and have lower educational attainment, lower income levels, and higher rates of divorce or widowhood. Notably, they were less likely to be regular alcohol consumers.

**Table 1:** Characteristics of 16,645 US adults ≥ 20 years, stratified by Stroke, the National Health and Nutrition Examination Survey 1999 to 2018.

Variable	Total	No stroke	Stroke	*P*
Age, year	46.81 ± 0.24	46.33 ± 0.24	63.88 ± 0.77	<.0001
Age, strata				
<60	10,956(75.59)	10,798(76.76)	158 (34.53)	<.0001
≥60	5689(24.41)	5244(23.24)	445 (65.47)	
Sex				
Female	8325(50.93)	8019(50.74)	306 (57.34)	.02
Male	8320(49.07)	8023(49.26)	297 (42.66)	
Race				
Non-Hispanic White	7946(70.92)	7626(70.89)	320 (72.05)	.01
Non-Hispanic Black	3187(10.45)	3039(10.35)	148 (13.79)	
Mexican American	2850(7.59)	2783(7.68)	67 (4.26)	
Other race	2662(11.04)	2594(11.08)	68 (9.91)	
Education levels				
<9th grade	1751(5.17)	1659(5.06)	92 (8.92)	< .0001
9–11th grade (includes 12th grade with no diploma)	2333(10.72)	2210(10.52)	123 (17.64)	
High school graduate or GED or equivalent	3844(24.03)	3689(23.85)	155 (30.28)	
Some college or aa degree	4867(31.30)	4707(31.42)	160 (27.29)	
College graduate or above	3850 (28.78)	3777(29.15)	73 (15.88)	
Poverty				
Below poverty (<1.3)	4762(19.84)	4543(19.59)	219 (28.43)	<.0001
Above poverty (≥1.3)	11,883(80.16)	11,499(80.41)	384 (71.57)	
Marital status				
Never married	2806(17.27)	2769(17.64)	37 (4.20)	<.0001
Married/living with partner	10,273(65.17)	9920(65.14)	353 (66.33)	
Widowed/divorced/separated	3566(17.55)	3353(17.21)	213 (29.48)	
Smoking				
Never	8941(53.38)	8707(53.83)	234 (37.49)	<.0001
Former	4306(25.59)	4071(25.23)	235 (38.29)	
Now	3398(21.03)	3264(20.94)	134 (24.22)	
Drinking				
Never	2212(10.62)	2115(10.52)	97 (14.10)	<.0001
Former	2891(14.27)	2677 (13.71)	214 (33.97)	
Now	11,542(75.11)	11,250(75.77)	292 (51.94)	
Coronary heart disease				
No	15,962(96.51)	15,465(96.91)	497 (82.23)	<.0001
Yes	683 (3.49)	577 (3.09)	106 (17.77)	
BMI, continuous	28.77 ± 0.08	28.73 ± 0.09	30.14 ± 0.46	.004
BMI, strata				
Low	3821 (24.85)	3722 (25.06)	99 (17.56)	.01
High	12,824(75.15)	12,320(74.94)	504 (82.44)	
Diabetes				
No	13,636(86.43)	13,264(86.98)	372 (66.96)	<.0001
Yes	3009 (13.57)	2778 (13.02)	231 (33.04)	
Hypertension				
No	9619 (63.21)	9488 (64.30)	131 (24.78)	<.0001
Yes	7026 (36.79)	6554 (35.70)	472 (75.22)	
MDS				
0	7388 (44.24)	7296 (45.01)	92 (16.86)	<.0001
1	5578 (35.94)	5415 (36.16)	163 (27.98)	
2	2512 (14.19)	2310 (13.70)	202 (31.45)	
3	960 (4.66)	841 (4.22)	119 (20.07)	
4	198 (0.94)	172 (0.86)	26 (3.55)	
5	9 (0.04)	8 (0.04)	1 (0.07)	
Income	3.05 ± 0.03	3.07 ± 0.03	2.44 ± 0.09	<.0001
TC, mmol/L	194.16 ± 0.48	194.30 ± 0.50	189.39 ± 2.24	.04
HDL-c, mmol/L	53.96 ± 0.21	53.96 ± 0.21	53.64 ± 0.99	.74
LDL-c, mmol/L	116.04 ± 0.40	116.23 ± 0.41	109.14 ± 1.96	<.001
TGs, mmol/L	120.89 ± 0.81	120.54 ± 0.82	133.15 ± 3.42	<.001
ALT, U/L	25.64 ± 0.22	25.70 ± 0.23	23.68 ± 0.92	.04
AST, U/L	25.07 ± 0.16	25.08 ± 0.16	24.64 ± 0.51	.43

All data is presented as number (weighted percentage %) or mean ± standard deviation.

ALT = alanine transaminase, AST = aspartate transaminase, BMI = body mass index, HDL-C = high-density lipoprotein cholesterol, LDL-c = low-density lipoprotein cholesterol, MDS = magnesium depletion score, TC = total cholesterol, TGs = triglycerides.

Clinically, stroke patients had significantly higher BMI, alanine aminotransferase (ALT), aspartate aminotransferase (AST), triglycerides (TG), TC, low-density lipoprotein cholesterol (LDL-C), and MDS scores. Prevalences of hypertension, diabetes, and CHD were also substantially higher in this group.

When analyzing MDS categories (0–5), the stroke group showed a significantly lower proportion of individuals with MDS = 0 (16.86% vs 45.01% in the non-stroke group) and a significantly higher proportion of individuals with MDS ≥ 2 (*P* <.05 for all), indicating a strong association between higher MDSs and stroke history.

## 3.2. Univariate logistic regression

Table 2 presents the univariable logistic regression results evaluating the association between various factors and stroke risk. A significant association was observed between MDS and stroke risk, with an odds ratio (OR) of 2.17 (95% CI: 1.98–2.39), indicating a 117% increased risk of stroke per 1-unit increase in MDS. When MDS was categorized into 6 levels, stroke risk showed a progressive increase with higher MDS scores. Using MDS = 0 as the reference group, the ORs (95% CIs) for each MDS category were: MDS = 1: 2.07 (1.52–2.80), MDS = 2: 6.13 (4.48–8.38), MDS = 3: 12.68 (8.65–18.59), and MDS = 4: 10.99 (6.16–19.63). All MDS levels from 1 to 4 were significantly associated with increased stroke risk (*P* <.05 for all).

**Table 2:** Univariate logistics regression analysis of the association between magnesium depletion score and stroke.

Charcter	OR (95% CI)	*P*
Age, year	1.07 (1.06–1.08)	<.0001
Age, strata		
<60	Reference	Reference
≥60	6.26 (4.93–7.95)	<.0001
Sex		
Male	0.77 (0.61–0.96)	.02
Female	Reference	Reference
Race		
Non-Hispanic White	Reference	Reference
Non-Hispanic Black	1.31 (1.04–1.66)	.02
Mexican American	0.55 (0.40–0.74)	<.001
Other Race	0.88 (0.59–1.30)	.52
Education levels		
<9th grade	Reference	Reference
9–11th grade (includes 12th grade with no diploma)	0.95 (0.67–1.35)	.78
High school graduate or GED or equivalent	0.72 (0.52–0.99)	.72
Some college or aa degree	0.49 (0.35–0.70)	.49
College graduate or above	0.31 (0.21–0.47)	.31
BMI,continuous	1.03 (1.01–1.04)	.001
BMI, Strata		
Low	Reference	Reference
High	1.57 (1.13–2.17)	.01
Smoking		
Never	Reference	Reference
Former	2.18 (1.77–2.68)	<.0001
Now	1.66 (1.24–2.22)	<.001
Drinking		
Never	Reference	Reference
Former	1.85 (1.29–2.64)	<.001
Now	0.51 (0.37–0.70)	<.0001
Hypertension		
No	Reference	Reference
Yes	5.47 (4.27–6.99)	<.0001
Diabetes		
No	Reference	Reference
Yes	3.30 (2.60–4.18)	<.0001
Marital status		
Never married	Reference	Reference
Married/living with partner	4.28 (2.85–6.44)	<.0001
Widowed/Divorced/Separated	7.20 (4.71–11.01)	<.0001
Coronary heart disease		
No	Reference	Reference
Yes	6.78 (5.03–9.15)	<.0001
Poverty		
Below poverty (<1.3)	Reference	Reference
Above poverty (≥1.3)	0.61 (0.50–0.76)	<.0001
MDS	2.17 (1.98–2.39)	<.0001
0	Reference	Reference
1	2.07 (1.52–2.80)	<.0001
2	6.13 (4.48–8.38)	<.0001
3	12.68(8.65–18.59)	<.0001
4	10.99(6.16–19.63)	<.0001
5	5.32 (0.63–44.77)	.12
Income	0.79 (0.74–0.84)	<.0001
ALT, U/L	0.99 (0.98–1.00)	.14
AST, U/L	1.00 (0.99–1.00)	.53
TGs, mmol/L	1.00 (1.00–1.00)	<.001
TC, mmol/L	1.00 (0.99–1.00)	.05
HDL-c, mmol/L	1.00 (0.99–1.01)	.74
LDL-c, mmol/L	0.99 (0.99–1.00)	.001

ALT = alanine transaminase, AST = aspartate transaminase, BMI = body mass index, HDL-C = high-density lipoprotein cholesterol, LDL-c = low-density lipoprotein cholesterol, MDS = magnesium depletion score, OR = odds ratio, TC = total cholesterol, TGs = triglycerides.

## 3.3. Multivariable logistic regression

Table 3 presents the multivariable logistic regression results assessing the association between MDS and stroke risk across 4 models. In the crude (unadjusted) model, higher MDS was significantly associated with increased stroke risk, with an OR (95% CI) of 2.17 (1.98–2.39) per 1-unit MDS increase. Categorical analysis showed a significant upward trend in stroke risk with increasing MDS levels (*P* for trend <.0001).

**Table 3:** Multivariable logistics regression analysis of the association between magnesium depletion score and stroke.

Character	Model	*P*	Model1	*P*	Model2	*P*	Model3	*P*
	Crude model							
	95% CI		95% CI		95% CI		95% CI	
Total	2.17 (1.98–2.39)	**<.0001**	1.43 (1.26–1.62)	**<.0001**	1.38 (1.22–1.56)	**<.0001**	1.27 (1.12–1.44)	**<.001**
0	Reference		Reference		Reference		Reference	
1	2.07 (1.52**–**2.80)	**<.0001**	1.20 (0.84**–**1.72)	.31	1.19 (0.84**–**1.70)	.32	1.16 (0.81**–**1.65)	.42
2	6.13 (4.48**–**8.38)	**<.0001**	2.24 (1.50**–**3.35)	**<.001**	2.11 (1.41**–**3.15)	**<.001**	1.83 (1.21**–**2.76)	**.004**
3	12.68(8.65**–**18.59)	**<.0001**	3.18 (1.94**–**5.21)	**<.0001**	2.85 (1.74**–**4.68)	**<.0001**	2.26 (1.38**–**3.72)	**.001**
4	10.99(6.16**–**19.63)	**<.0001**	2.46 (1.32**–**4.61)	**.01**	2.14 (1.15**–**3.99)	**.02**	1.66 (0.87**–**3.17)	.12
5	5.32 (0.63**–**44.77)	.12	1.66 (0.22**–**12.41)	.62	1.38 (0.18**–**10.93	.76	1.06 (0.13**–**8.87)	.96
***P* for trend**		**<.0001**		**<.0001**		**<.0001**		**<.001**

Crude model: no adjustments made for confounding factors. Model 1: adjustments made for age, sex, race, marital status, income,and education levels. Model 2: age, sex, race, marital status, income, education levels, BMI, ALT, AST, TGs, TC, HDL-c, LDL-c, smoking, and drinking. Model 3: age, sex, race, marital status, income, education levels, BMI, ALT, AST, TGs, TC, HDL-c, LDL-c, smoking, drinking, diabetes, hypertension, and coronary heart disease. Bold font indicates the corresponding *P* values are **<**.05, signifying statistical significance.

ALT = alanine transaminase, AST = aspartate transaminase, BMI = body mass index, HDL-C = high-density lipoprotein cholesterol, LDL-c = low-density lipoprotein cholesterol, MDS = magnesium depletion score, OR = odds ratio, TC = total cholesterol, TGs = triglycerides.

In Model 1, after adjusting for age, sex, race/ethnicity, marital status, income, and education, the association between MDS and stroke risk was slightly attenuated but remained significant (OR = 1.43, 95% CI: 1.26–1.62 per 1-unit increase). Compared to the MDS = 0 group, participants with MDS = 2 to 4 had significantly higher stroke risk.

Model 2, further adjusting for BMI, ALT, AST, TG, HDL-C, TC, LDL-C, smoking, and alcohol consumption, showed the OR per 1-unit MDS increase decreased to 1.38 (1.22–1.56), with persistent significance. Individuals with MDS = 2 to 4 still had significantly elevated stroke risk compared to MDS = 0.

In Model 3, fully adjusted for diabetes, hypertension, and CHD, the OR further declined to 1.27 (1.12–1.44) per 1-unit MDS increase. Notably, participants with MDS = 2 to 3 remained significantly associated with higher stroke risk after adjusting for all covariates (*P* <.05 for all).

## 3.4. Restricted cubic spline analysis

**Figure 2.**
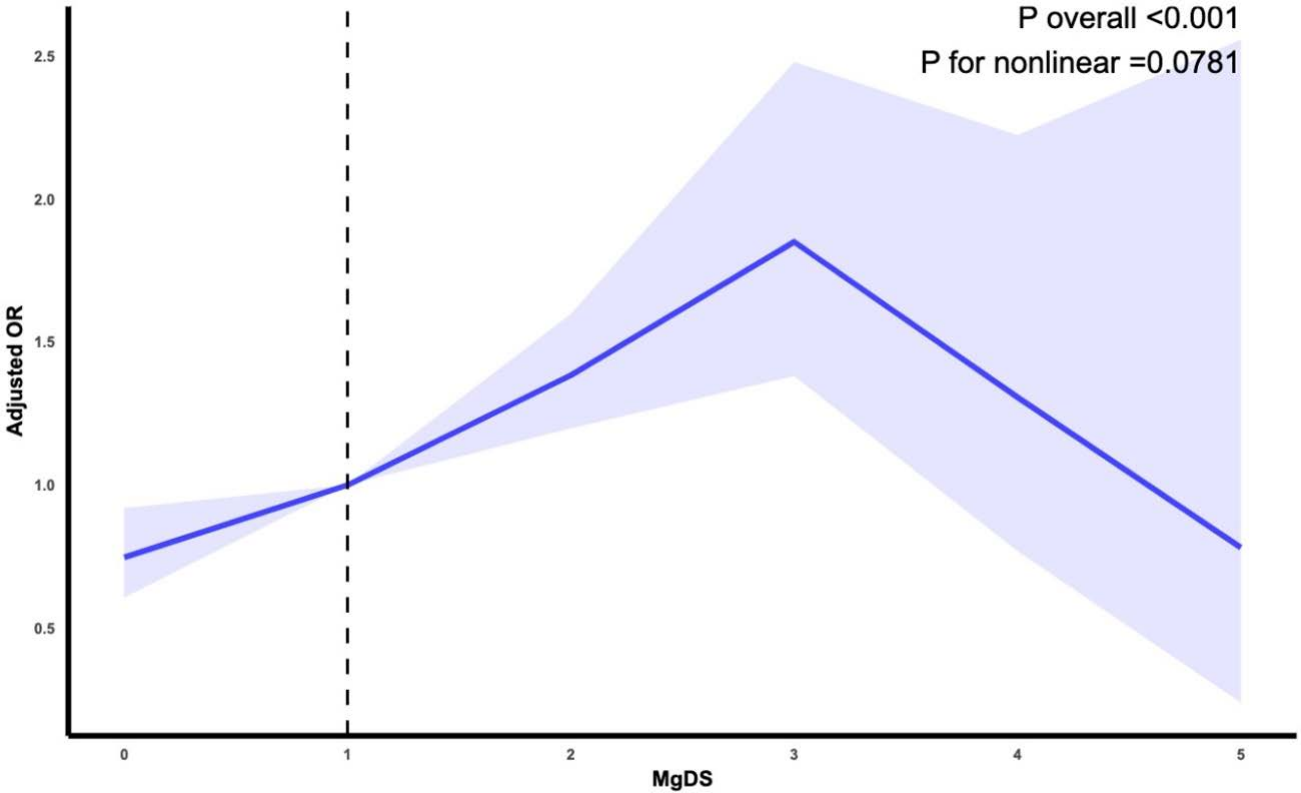
Nonlinear associations between MDS and risk of Stroke. The RCS plot between MDS and Stroke. The x-axis represents MDS, while the y-axis represents the OR and the 95% CI for Stroke The dashed line indicates an OR of 1, which represents no association between MDS and stroke risk. The model adjusted by age, sex, race, body mass index, marital status, income, education level, alanine transaminase, aspartate transaminase, triglycerides, total cholesterol, high-density lipoprotein cholesterol, low-density lipoprotein cholesterol, corrected smoking, drinking, diabetes, hypertension and coronary heart disease. CI = confidence interval, MDS = magnesium depletion score, OR = odds ratio, RCS = restricted cubic spline.

Figure 2 illustrates the results of the RCS analysis, visually depicting the relationship between MDS and stroke risk. As MDS increased, the OR gradually rose, peaking at approximately MDS = 3, after which it stabilized and showed a slight decline. The *P*-value for nonlinearity was >.05, indicating no significant nonlinear relationship between MDS and stroke risk. Overall, the findings suggest a linear positive association between higher MDS and increased stroke risk, with no apparent threshold effect.

## 3.5. Stratified analysis of the association between MDS and stroke

This study evaluated the association between MDS and stroke risk across subgroups using multivariable logistic regression, adjusting for potential confounders (Table 4). Subgroups were stratified by age, sex, race/ethnicity, marital status, poverty level, education, BMI, alcohol consumption, and smoking status.

**Table 4:** Subgroup analysis for the association between magnesium depletion score and stroke.

Character	OR (95% CI)	*P*	*P* for interaction
Age,Strata			
<60	1.19 (0.94,1.49)	.14	.82
≥60	1.33 (1.17,1.52)	**<.0001**	
Sex			
Female	1.25 (1.04,1.50)	**.02**	.25
Male	1.31 (1.11,1.54)	**.002**	
Race			
Non-Hispanic White	1.27 (1.07, 1.50)	**.01**	.72
Non-Hispanic Black	1.45 (1.20,1.74)	**<.001**	
Other race (including multi-racial and other Hispanic)	1.24 (0.88, 1.75)	.23	
Mexican American	1.11 (0.86, 1.43)	.42	
Marital status			
Never married	1.77 (1.03, 3.04)	**.04**	.41
Married/living with partner	1.25 (1.07,1.46)	**.01**	
Widowed/divorced/separated	1.30 (1.08,1.56)	**.01**	
Poverty			
Below poverty (<1.3)	1.16 (0.90,1.49)	.25	.12
Above poverty (≥1.3)	1.33 (1.16,1.53)	**<.0001**	
Education levels			
<9th grade	1.07 (0.82, 1.39)	.63	.31
9–11th grade (includes 12th grade with no diploma)	1.28 (0.98,1.67)	.07	
High school graduate or GED or equivalent	1.51 (1.20,1.89)	**<.001**	
Some college or aa degree	1.15 (0.92,1.45)	.22	
College graduate or above	1.18 (0.88, 1.58)	.27	
BMI Strata			
Low	1.25 (0.93, 1.66)	.13	.37
High	1.28 (1.13,1.46)	**<.001**	
Drinking			
Never	1.47 (1.11, 1.93)	**.01**	.13
Former	1.27 (1.03, 1.57)	**.03**	
Now	1.26 (1.07,1.48)	**.01**	
Smoking			
Never	1.38 (1.14,1.66)	**<.001**	.09
Former	1.30 (1.06,1.58)	**.01**	
Now	1.15 (0.92,1.45)	.23	

Subgroup analysis for the association between MDS and stroke weighted univariate logistic regression was used for subgroup analysis. Adjustments were made for education levels,hypertension, diabetes,coronary heart disease, ALT, AST, TGs, TC, HDL-c, LDL-c. Bold font indicates the corresponding *P* values are **<**.05, signifying statistical significance.

ALT = alanine transaminase, AST = aspartate transaminase, BMI = body mass index, HDL-C = high-density lipoprotein cholesterol, LDL-c = low-density lipoprotein cholesterol, MDS = magnesium depletion score, OR = odds ratio, TC = total cholesterol, TGs = triglycerides.

Results showed MDS was significantly associated with stroke risk in most subgroups, with ORs ranging from 1.07 to 1.77 per 1-unit MDS increase, indicating a consistent positive association across populations. While the association’s strength varied slightly – for example, stronger effects were observed in individuals aged ≥ 60 years, never smokers/nondrinkers, and those with higher BMI – all interaction terms had *P* >.05, indicating no statistically significant subgroup differences.

## 4. Discussion

This study analyzed the relationship between the MDS and stroke risk using NHANES data from 1999 to 2018. The findings revealed that a higher MDS, indicative of significant urinary magnesium loss, was independently associated with an increased risk of stroke. Additionally, a significant positive linear relationship was observed between MDS and stroke risk, with no apparent threshold effect. Notably, even after adjusting for sociodemographic characteristics, lifestyle factors, and cardiovascular metabolic confounders, an increase in MDS remained significantly associated with a higher risk of stroke. These findings further underscore the critical role of magnesium in stroke development. Notably, when the continuous MDS is categorized, the MDS = 1 category may have insufficient statistical power, or its effect size might be too weak to reach statistical significance. However, the RCS analysis reveals an overall dose-response trend across the entire MDS range, suggesting that even if the association with MDS = 1 is not statistically significant, the risk may have already begun to increase. This positive trend becomes more evident when MDS is treated as a continuous variable.

This study identified a significant positive association between MDS, a newly developed assessment tool, and stroke risk. These findings are supported by multiple clinical studies. A systematic review and meta-analysis evaluated the relationship between higher magnesium intake and reduced stroke risk, including 15 publications covering 18 cohort studies. The results indicated that, compared to the lowest intake group, an additional 100 mg of daily magnesium intake was associated with an 11% reduction in overall stroke risk (OR = 0.89, 95% CI: 0.83–0.94, *P* <.001), with further analyses confirming a dose-dependent inverse relationship.^[20]^ A randomized, double-blind, placebo-controlled clinical trial demonstrated that oral magnesium supplementation significantly reduced low-density lipoprotein cholesterol (LDL-C), TC, triglycerides, and blood glucose levels, all closely associated with stroke risk.^[21]^ However, some studies have reported divergent findings: for example, certain studies suggest magnesium intake is inversely associated with stroke risk in women (with postmenopausal hormonal factors potentially influencing this sex difference), while no such effect is observed in males.^[22]^ Other studies report no significant association, possibly due to insufficient magnesium intake among participants limiting the manifestation of health benefits.^[23]^ Although these studies adjusted for age, sex, and BMI, genetic susceptibility and other unmeasured factors may still influence results.^[24]^ A common limitation is their reliance on serum magnesium levels or dietary intake alone, which reflect only partial magnesium status and may not accurately capture systemic or intracellular stores, potentially leading to inconsistent conclusions. Thus, developing more comprehensive assessment tools like MDS – which integrates multiple factors to evaluate magnesium status – is crucial for advancing research and clinical applications.

Given that stroke patients frequently exhibit atherosclerosis, neuronal damage, and cerebral thrombus formation,^[25]^ this study hypothesizes that magnesium deficiency contributes to stroke via multiple mechanisms. Magnesium deficiency enhances oxidative stress and activates inflammatory pathways (e.g., NF-κB signaling), disrupting vascular bioactive molecule synthesis (e.g., NO, PGI₂) and inducing endothelial dysfunction and atherosclerosis.^[26]^ It also promotes hypertriglyceridemia and lipoprotein metabolism disorders, accelerating atherosclerotic progression.^[27]^ Neuroprotectively, magnesium regulates NMDA receptor activity to prevent calcium overload and excitotoxicity; deficiency weakens this mechanism, exacerbating neuronal damage.^[28]^ As a cofactor for antioxidant enzymes (e.g., superoxide dismutase), magnesium deficiency increases reactive oxygen species production, intensifying oxidative stress and neuroinflammation.^[29]^ In the coagulation system, magnesium deficiency enhances platelet aggregation and alters coagulation factor activity, promoting thrombus formation.^[30,31]^ While precise mechanisms require further elucidation, existing evidence strongly supports magnesium deficiency as a contributor to stroke pathogenesis.

Clinically, serum magnesium levels have limitations: accounting for only ~1% of total body magnesium, they are influenced by transient homeostasis and fail to reflect bone/soft tissue reserves.^[32,33]^ In conditions like metabolic syndrome, normal serum levels may coexist with intracellular depletion.^[34]^ The magnesium tolerance test, though more accurate, requires 24-hour urine collection and intravenous infusion, limiting clinical feasibility due to sampling challenges and resource-intensive monitoring.^[35,36]^ By contrast, MDS is a noninvasive, practical tool integrating renal reabsorption capacity, dietary intake, medication use (PPIs, diuretics), and chronic disease status (e.g., CKD) to comprehensively assess systemic magnesium levels. Studies show MDS outperforms serum magnesium and magnesium tolerance test in predicting magnesium deficiency-related risks (e.g., cardiovascular disease, inflammation, mortality),^[37]^ effectively identifying high-risk individuals for early chronic disease intervention.^[38]^

Our study demonstrates that high MDS is significantly associated with increased stroke risk among U.S. adults. MDS components – alcohol consumption, PPI use, diuretic use, and CKD – are each linked to stroke: alcohol increases magnesium excretion and cerebrovascular risk^[39]^; long-term PPI/diuretic use induces hypomagnesemia^[40,41]^; CKD disrupts magnesium metabolism, promoting arteriosclerosis and inflammation.^[42]^ MDS thus captures the cumulative impact of these factors on stroke pathogenesis.

The study’s strengths include a large NHANES dataset (16,645 participants) with broad representativeness across age, sex, race, and socioeconomic status, along with rigorous multistage sampling and statistical methods (logistic regression, RCS modeling) that account for multiple confounders. Limitations include reliance on self-reported stroke data (potential information bias), MDS’s inability to directly measure magnesium metabolism mechanisms, unmeasured confounders (e.g., genetics), and cross-sectional design precluding causal inference. Despite these, MDS offers a robust approach to identifying magnesium deficiency-related stroke risk, informing public health and clinical strategies.

In conclusion, this study establishes MDS as a valuable marker for stroke risk assessment, highlighting the importance of comprehensive magnesium status evaluation in stroke prevention. The findings support further research into MDS-guided interventions to mitigate magnesium deficiency and reduce stroke burden. Notably, these findings do not entirely rule out potential population-specific effects of MDS. Future research should explore biological mechanisms to clarify whether these variations hold clinical significance. Overall, MDS serves as an independent and robust risk factor for stroke, unaffected by sociodemographic or lifestyle factors.

## 5. Conclusion

This study demonstrates a significant association between the MDS and stroke risk, with consistent findings across subgroup analyses, providing robust evidence for clinical management, preventive strategies, and public health interventions. By validating MDS as a comprehensive tool for assessing magnesium status, this research fills a critical gap by being the first to examine the link between magnesium deficiency and stroke risk in the general population using this simple, practical metric.

The ability of MDS to identify community-dwelling individuals at risk of magnesium deficiency enables targeted interventions to reduce stroke risk. Specific strategies include: reducing alcohol consumption, optimizing the use of PPIs and diuretics, managing CKD, and increasing dietary magnesium intake or supplementation. These modifiable interventions highlight the potential of nutritional and lifestyle adjustments in stroke prevention, offering pragmatic approaches to lower population-level stroke burden.

In summary, this study underscores MDS as a valuable tool for integrating magnesium status assessment into clinical practice, advocating for its use in early risk identification and personalized preventive care.

## Author contributions

**Conceptualization:** Li-Chao Zhang.

**Data curation:** Li-Chao Zhang, Zhi-Qiang Ye, Wen-Liang Shuai, Chen-Qi He.

**Formal analysis:** Li-Chao Zhang.

**Investigation:** Li-Chao Zhang, Wen-Liang Shuai, Li-Min Zhuang.

**Methodology:** Wen-Liang Shuai, Xu-Ying Yu.

**Project administration:** Chen-Qi He, Li-Min Zhuang.

**Resources:** Chen-Qi He.

**Software:** Zhi-Qiang Ye, Chen-Qi He, Xu-Ying Yu.

**Supervision:** Xu-Ying Yu.

**Validation:** Zhi-Qiang Ye.

**Visualization:** Li-Min Gong.

**Writing – original draft:** Li-Chao Zhang, Zhi-Qiang Ye.

**Writing – review & editing:** Li-Min Gong.
